# Dietary Intake and Arterial Stiffness in Children and Adolescents: A Systematic Review

**DOI:** 10.3390/nu15092092

**Published:** 2023-04-26

**Authors:** Allanah Leed, Emma Sheridan, Brooke Baker, Sara Bamford, Elana Emmanouilidis, Fletcher Stewart, Kristen Ostafe, Mustafa Sarwari, Karen Lim, Miaobing Zheng, Sheikh Mohammed Shariful Islam, Kristy A. Bolton, Carley A. Grimes

**Affiliations:** 1 School of Exercise and Nutrition Sciences, Deakin University, Geelong 3216, Australia; 2 Institute for Physical Activity and Nutrition, School of Exercise and Nutrition Sciences, Deakin University, Geelong 3216, Australia

**Keywords:** dietary intake, dietary pattern, dietary supplement, omega-3 supplementation, vitamin D supplementation, arterial stiffness, pulse wave velocity, aortic stiffness, child, adolescent

## Abstract

Arterial stiffness is a risk factor for cardiovascular disease that is affected by diet. However, research understanding how these dietary risk factors are related to arterial stiffness during childhood is limited. The purpose of this review was to determine whether various dietary factors were associated with arterial stiffness in the pediatric population. Five databases were systematically searched. Intervention studies, cross-sectional and cohort studies were included that investigated nutrient or food intake and outcomes of arterial stiffness, primarily measured by pulse wave velocity (PWV) and augmentation index (AIx), in the pediatric population (aged 0–18 years). A final 19 studies (six intervention and 13 observational) were included. Only two intervention studies, including a vitamin D and omega-3 supplementation trial, found protective effects on PWV and AIx in adolescents. Findings from observational studies were overall inconsistent and varied. There was limited evidence to indicate a protective effect of a healthy dietary pattern on arterial stiffness and an adverse effect of total fat intake, sodium intake and fast-food consumption. Overall, results indicated that some dietary factors may be associated with arterial stiffness in pediatric populations; however, inconsistencies were observed across all study designs. Further longitudinal and intervention studies are warranted to confirm the potential associations found in this review.

## 1. Introduction

Globally, cardiovascular disease (CVD) is the largest contributor to non-communicable diseases, with the total number of deaths from CVD steadily increasing from 12.1 million in 1990 to 18.6 million in 2019 [1]. CVD is considered a largely preventable disease, as a plethora of the associated risk factors are considered modifiable, including smoking, hypertension and poor nutrition [2]. Importantly, early prevention is fundamental to reducing CVD prevalence over the lifespan, as subclinical changes in cardiovascular structure and function can commence during childhood [3,4].

Arterial stiffness is increasingly used as a pre-clinical measure of CVD. Decreased elasticity of arterial walls represents one of the earliest markers of damage to the vascular system [5]. Among adults, arterial stiffness is an independent predictor of cardiovascular events and mortality and all-cause mortality [6]; among adolescents and young adults, it has been associated with target organ damage [7]. While various methods are available to determine the degree of arterial stiffness, the gold standard is pulse wave velocity (PWV) [8,9]. Stiffening of the arteries naturally progresses with age over the lifespan; however, the process can be accelerated with certain conditions, such as obesity [10,11] and via environmental factors [9].

A few systematic reviews have investigated whether a relationship is evident between dietary intake and arterial stiffness, with the majority focusing on single nutrients and all in adult populations [12,13,14,15]. For instance, an earlier systematic review of randomized control trials (RCTs) conducted in 2012 explored whether nutrient and dietary interventions reduced arterial stiffness in adults [12]. Findings from this review showed some evidence of an effect via supplementation with omega-3 fatty acids and soy isoflavones [12]. A more recent systematic review and meta-analysis of RCTs published in 2018 investigated the effect of dietary sodium restriction in adults on arterial stiffness and found that decreased consumption was linked to reduced arterial stiffness [14]. Further, an umbrella review of systematic reviews and meta-analyses were conducted in 2019, which investigated the effect of vitamin C supplementation towards cardiovascular markers, including arterial stiffness [13]. Ultimately, no effects were exhibited between vitamin C supplementation and arterial stiffness [13].

While there is emerging literature examining the relationship between nutrient and dietary intake [16,17,18], including nutrient supplementation [19], and measures of arterial stiffness among children, to date, no systematic reviews have been conducted within the pediatric population. Understanding how modifiable lifestyle factors, such as dietary intake, are related to arterial stiffness during childhood is important for the development and implementation of dietary strategies to protect cardiovascular health. Therefore, the aim of this systematic review was to determine whether dietary factors, including patterns, nutrients and food intake, were associated with arterial stiffness within the pediatric population.

## 2. Materials and Methods

This systematic review followed the Preferred Reporting Items for Systematic Reviews and Meta-Analysis (PRISMA) guidelines [20] and was registered with the International Prospective Register of Systematic Reviews (PROSPERO; registration number CRD42022315443).

### 2.1. Literature Search

A literature search was performed across five databases, including MEDLINE Complete and CINAHL Complete (via EBSCOhost platform), Scopus, Cochrane Library and Embase (via Elsevier), identifying studies published from inception to 4 March 2022. The Population; Intervention/Exposure; Comparison; Outcome (PICO) framework was utilized to develop the research question and search strategy. Participants: Children and adolescents aged 0–18 years; Intervention/Exposure: Food, nutrient intake or dietary pattern; Comparison: for intervention studies, children with less exposure will be compared to the control group, e.g., an intervention group receiving a dietary supplement compared to control group not receiving supplementation; Outcomes: Indexes of arterial stiffness. These include pulse wave velocity (PWV), which, when measured at the carotid-femoral site, is considered the reference method for arterial stiffness [8,9], augmentation index (AIx) and arterial distensibility. PWV measures the speed (m/s) for the arterial pulse to travel between the carotid and femoral arteries, with higher values representing increased stiffness in the arteries [9]. AIx is an indirect measure that calculates the ratio between increased pressure of reflected wave and pulse pressure or simply the difference between systolic and diastolic blood pressure [21]. An increased AIx infers greater arterial stiffness.

The search strategy included three concepts related to the research aim: (1) dietary factors; (2) indexes of arterial stiffness; (3) population of interest. Search terms were supplemented with appropriate Medical Subject Headings (MeSH). Examples of search terms included: “dietary intake” OR “nutrient intake” OR Food* OR “dietary pattern” OR vitamin OR mineral (concept 1) AND “arterial stiff*” OR “pulse wave velocity” OR “vascular ageing” (concept 2) AND child* OR adolesce* OR pediatric (concept 3). The full search strategy across all databases can be found in supplementary file S1 (Supplementary Tables S1–S5). Reference lists of included studies were searched to identify any additional studies.

### 2.2. Eligibility Criteria

To be eligible for inclusion, studies were required to meet the following criteria: have used a cross-sectional, cohort or intervention study design; have investigated nutrient or food intake; and measured outcomes of arterial stiffness in children and/or adolescents.

Eligible studies were sourced from the aforementioned databases, being limited to English only and human studies, but with no limits on the date of publication. Conference abstracts were excluded. Studies that included participants older than 18 years of age were included if the mean age of the sample was <18 years. Any studies that included participants with a chronic or genetic disease (e.g., type 1 diabetes) were excluded, as the focus of the review was to determine the effects of dietary factors on generally healthy children and adolescents (i.e., not targeting any specific health condition). However, given the high prevalence of overweight and obesity in the general pediatric population, no restriction was placed on children with these conditions. Furthermore, understanding the potential effect of diet on arterial stiffness among children with obesity is important, given the increased risk of arterial stiffening within this group [11]. Studies that did not assess the exposure or have a dietary intervention, such as those monitoring effects of physical exercise, were excluded. Included indexes of arterial stiffness were PWV, AIx and carotid or brachial artery distensibility.

### 2.3. Data Management and Extraction

Articles obtained through the literature search were imported to EndNote, and then exported into Covidence, where duplicate records were removed. Screening at title and abstract level, followed by full-text review, was completed in duplicate by two independent reviewers. Any conflicts for article inclusion were resolved via discussion, and if necessary, a third reviewer was consulted. A data extraction table was created and piloted with two studies and refined to ensure all relevant data were collected. Data were then extracted from eligible studies independently by two reviewers. Extracted data included: aim, study design, sample characteristics, measures of exposure and outcome, intervention details (where relevant), statistical analysis (including covariate adjustment) and study findings. In all instances, the most adjusted model was preferentially extracted.

### 2.4. Quality Assessment

The Academy of Nutrition and Dietetics Quality Criteria Checklist was used to assess overall quality and risk of bias of included studies [22] independently by two reviewers. Any disagreement in assessment was resolved via discussion. The tool consists of four relevance questions that assessed significance related to the topic of interest and dietetic practice, followed by a further ten questions (each with sub-questions) that scrutinized the validity of the study, including participant selection, blinding, methods of measurement and statistical analysis [22]. Each question was answered with yes (Y), no (N), unclear (U) or not applicable (NA). Each study received an overall positive, neutral or negative quality rating based on validity question answers. A positive rating indicated a low risk of bias, a neutral score indicated moderate risk of bias and a negative score indicated high risk of bias. Studies allocated an ‘N’ for six or more validity questions were rated negative. Those with answers for validity questions 2, 3, 6 and 7 that were less than exceptionally strong were rated neutral. For studies with the majority of validity questions assigned ‘Y’ (including questions 2, 3, 6, 7), a positive rating was allocated [22].

### 2.5. Data Synthesis

Data were grouped according to the study design, and then further stratified based on the assessed dietary component (e.g., omega-3, vitamin D, dietary patterns, sodium). Due to the limited number of studies retrieved and heterogeneity in dietary exposures, indexes of arterial stiffness, covariate adjustment and methodologies used across studies, no meta-analysis was performed.

## 3. Results

### 3.1. Study Selection

**Figure 1:**
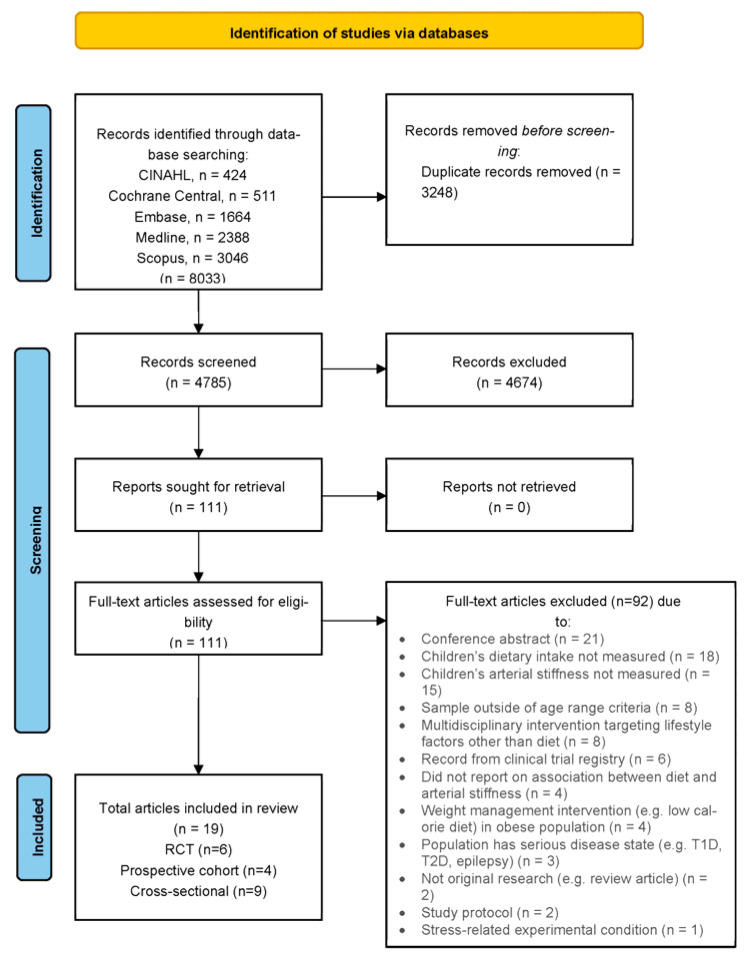
PRISMA Flow Chart. Preferred Reporting Items for Systematic Reviews and Meta-Analyses (PRISMA) Flowchart. T1D: type 1 diabetes; T2D: type 2 diabetes.

In total, 8033 articles were identified across five databases, 3248 of which were duplicates and removed (Figure 1). Following title and abstract screening, 111 articles were retrieved for full-text assessment, 92 of which were excluded, leaving 19 articles for inclusion. No further studies were identified following a screening of the reference list of the included studies.

### 3.2. Characteristics of Included Studies

Table 1 summarizes characteristics of included studies. We identified 6 intervention studies, including 4 parallel RCTs [19,23,24,25], 1 randomized cross-over trial [26], 1 3-y follow-up of a parallel RCT [27] and 13 observational studies, including 4 prospective cohorts [18,28,29,30] and 9 cross-sectional studies [16,17,31,32,33,34,35,36,37]. One of the cross-sectional studies [31] was comprised of baseline data from one of the included parallel RCTs [24]; two of the prospective cohort studies were based on the same birth cohort study of children, however, had two independent aims (e.g., The Generation R Study) [29,30].

Articles were published between 2012 and 2022. Ten studies were conducted in Europe [17,24,26,28,29,30,31,32,34,36], three in the United States of America (USA) [16,19,23], four in Australia and New Zealand [18,27,35,37], one in India [25] and one study collected data across three countries (Angola, Brazil and Spain) [33]. A range of dietary components was assessed, including supplemental vitamin D (*n* = 3) [19,23,25], supplemental omega-3 fatty acids (*n* = 2) [26,27], supplemental milk proteins (*n* = 2) [24,31], infant feeding practices (*n* = 3), [28,29,33], dietary patterns (*n* = 6) [17,18,32,34,35,36], macronutrient intake (*n* = 2) [30,31], sodium intake (*n* = 1) [16] and consumption of specific food groups (*n* = 3) [17,31,37], such as fast food and sugar-sweetened beverages. The measures used to assess dietary intake varied across studies. Within intervention studies, compliance with supplements was assessed via tablet counting [19,23,26,27] or self-reported diaries [24], combined with biochemical measures (e.g., plasma 25(OH)D [19,23,25], serum omega-3 [26,27], or serum urea-Nitrogen [24]). Within observational studies, six used food frequency questionnaires (FFQ) [17,30,34,35,36,37], three used self-reported questionnaires assessing infant feeding practices [28,29,33], one used 7-day food records [28], one used 3-day food records [16], two used a 4-day food checklist [18,31], one used 24-h diet recall [36], one used the KIDMED 16-item questionnaire [32] and two studies used multiple measures [28,36]. The most commonly used index of arterial stiffness was PWV (*n* = 18), with most of these studies (*n* = 17) reporting carotid-femoral PWV (cf-PWV). Seven studies reported Aix, one study reported carotid arterial distensibility [27] and one study reported brachial artery distensibility [16] (Table 1). The general characteristics of the 19 included studies are shown in Table 2 and Table 3.

### 3.3. Quality Assessment

Overall, based on the Academy of Nutrition and Dietetics Quality Criteria Checklist, eight studies were rated as positive [18,19,23,27,28,29,35,37] and eleven as neutral [16,17,24,25,26,30,31,32,33,34,36] (Supplementary Table S6). When considering study design, prospective cohort studies tended to be deemed high quality (e.g., positive rating for 3/4 studies), half of the intervention studies were deemed high quality and half neutral and the majority of the cross-sectional studies (7/9) were deemed neutral.

### 3.4. Findings from Intervention Studies

Across the six intervention studies, sample sizes ranged from 25 to 225 participants, and intervention duration ranged from 12 weeks to 3 years. With the exception of one study conducted in 8-year-old children [27], the remaining were conducted in adolescents aged between 10 and 18 years. Four of the studies were completed in participants who were overweight or obese [19,24,25,26] (Table 4).

#### 3.4.1. Vitamin D Supplementation

Three RCTs examined the effect of vitamin D supplementation on PWV [19,23,25]. All studies used a lower dose of vitamin D in the active control, and the dosage in the intervention group ranged from 2000 IU/d of vitamin D3 to 120,000 IU/d. All studies showed increases in plasma 25(OH)D within the intervention group vs. control at the end of the intervention period. Two of these studies, both completed with adolescents who were overweight or obese, found no effect of vitamin D supplementation on cf-PWV following a 6- or 12-month intervention [19,25]. Conversely, the third study completed in apparently healthy African-American adolescents found an improvement in cf-PWV following vitamin D supplementation for a 16-week period [23]; however, there was no change in carotid-radial PWV or carotid-distal PWV.

#### 3.4.2. Omega-3 Fatty Acid Supplementation

Two intervention studies examined the effect of omega-3 supplementation on arterial stiffness [26,27]. The first randomized cross-over trial, completed in obese adolescents, showed no effect on carotid-radial PWV following 3 months of 1.2 g of omega-3 supplementation; however, there was an improvement in AIx [26]. Similarly, a 3-year follow-up of a previous 5-year parallel RCT showed no difference in brachial-PWV measured at 8 years of age when comparing children who had consumed a diet supplemented with omega-3 fatty acids and a control diet for the first 5 years of life [27]. In addition, this study found no effect on AIx or carotid artery distensibility.

#### 3.4.3. Milk Protein Supplementation

The final parallel RCT, which examined the effect of different types of milk proteins (e.g., supplementing diets with skimmed milk and casein and whey-based protein drinks) found the consumption of these drinks over a 12-week period resulted in no change in cf-PWV or AIx among overweight and obese adolescents when compared to a pretest control group or water group [24].

### 3.5. Findings from Observational Studies

#### 3.5.1. Prospective Cohort Studies

Across the four prospective cohort studies, the follow-up period ranged from 6 to 10 years and included between 93 and 4024 participants [18,28,29,30]. All were based on population birth cohorts.

#### 3.5.2. Cross-Sectional Studies

The majority of cross-sectional studies (6/9) were based on community samples of children recruited from schools, including elementary [17,33,34,35] and secondary schools [32,36] or a combination of both [34]. One of these school-based studies specifically targeted the recruitment of obese children [34]. Another cross-sectional study was completed on adolescents who were overweight or obese and recruited for an intervention study (reported above) [24,31]. The sample size of cross-sectional studies ranged from 75 to 1780 participants (Table 5).

#### 3.5.3. Infant Feeding Practices

##### Prospective Cohort Studies

Two prospective cohort studies examined infant feeding practices (breastfeeding or introduction to solids) [28,29] with mixed findings reported. For breastfeeding, one study reported a protective effect [29], while the other reported a negative effect [28]. In a large study, (*n* = 4024) de Jonge et al. [29] reported that at 6 years of age, cf-PWV was significantly lower in children who had been breastfed vs. never breastfed during the first 12 months of life. This study adjusted for a comprehensive list of maternal- and child-related covariates (Table 5). In contrast, in a much smaller study (*n* = 93), which included adjustment for child-related covariates only, Schack-Nielsen reported that longer duration of breastfeeding during the first 9 months of life was associated with significantly higher carotid-femoral-PWV measured at age 10 years; however, no association was observed for carotid-radial PWV [28]. The cohort study by de Jonge [29] also assessed the association between the timing of the introduction of solids and cf-PWV at 6 years of age and reported no association between these two variables.

##### Cross-Sectional Studies

One cross-sectional study examined the relationship between breastfeeding and arterial stiffness [33]. In this study of 520 children, no association was found between the duration of exclusive breastfeeding (retrospectively collected) and cf-PWV measured at age 9–10 years (adjusted for some child-related covariates) [33].

#### 3.5.4. Dietary Patterns

##### Prospective Cohort Studies

One prospective cohort study examined the association between dietary patterns and arterial stiffness [18]. In this cohort of children followed from age 4 to 15 years (*n* = 188), it was reported that among four empirically derived dietary pattern trajectories (‘unhealthy’, ‘moderately unhealthy’, ‘moderately healthy’ and ‘healthy’), none were associated with cf-PWV measured at 15 years of age (adjusted for maternal and child-related covariates) [18].

##### Cross-Sectional Studies

Five cross-sectional studies examined the association between dietary patterns and arterial stiffness [17,32,34,35,36]. Three of these studies reported null findings [17,34,35], and two reported inverse associations (e.g., protective) [32,36]. Empirically derived dietary patterns were identified and assessed in two of these studies [17,35]. Firstly, among Italian primary schoolchildren (*n* = 300), there were no associations identified between ‘healthy’ or ‘unhealthy’ dietary patterns and cf-PWV [17]. Secondly, among New Zealand primary schoolchildren (*n* = 389), there were no associations between identified dietary patterns characterized by ‘snacks’ or ‘fruit and vegetables’ and either cf-PWV or Aix [35]. Both of these cross-sectional studies adjusted for a range of child-related covariates, including physical activity (Table 5). The remaining three studies used scoring systems to assess adherence to either a Mediterranean dietary pattern (*n* = 2) [32,34] or a heart-healthy dietary pattern (*n* = 1) [36]. One study assessing adherence to the Mediterranean dietary pattern completed in elementary school students from Greece (*n* = 277) reported that higher adherence to this dietary pattern was significantly inversely associated with Aix (e.g., protective) (adjusted for child-related covariates) [32]. Conversely, in a small sample of Italian schoolchildren (*n* = 75), all with obesity, there was no association with adherence to a Mediterranean dietary pattern and cf-PWV (adjusted for child-related covariates) [34]. Finally, in a crude unadjusted analysis of Italian elementary schoolchildren (*n* = 387), cf-PWV was significantly lower (e.g., protective) among children with an ‘ideal’ heart-healthy diet score compared to children with a ‘not ideal’ score [36].

#### 3.5.5. Consumption of Specific Food Groups

##### Cross-Sectional Studies

Three cross-sectional studies assessed the association between consumption of specific food groups and arterial stiffness with mixed findings [17,31,37]. In a sample of Italian primary schoolchildren (*n* = 300), Giontella et al. [17] examined the association between 14 different foods/food groups and PWV. The only food found to be positively associated with cf-PWV was fast food. No associations were reported for cereals and tubers, vegetables, fruit, eggs, meat, dairy products, sweets, legumes, fish, nuts, extra virgin olive oil, animal-derived fat or seed oil [17]. Conversely, in a large population-based sample (*n* = 1780) of Australian children aged 11–12 years, it was reported that neither fast food nor sugar-sweetened beverage consumption was associated with cf-PWV [37]. Finally, in a sample of adolescents who were overweight or obese (*n* = 183), there was a trend whereby greater consumption of milk and yoghurt per day was associated with lower cf-PWV (*p* = 0.05) (e.g., protective), but no association was reported with Aix [31] All of these studies were adjusted for a range of child-related covariates (Table 5).

#### 3.5.6. Macronutrient Intake

##### Prospective Cohort Studies

One prospective cohort study assessed the association between macronutrient intake and arterial stiffness, with mixed findings across different macronutrients [30]. The prospective cohort study was based on a population sample of children (*n* = 2427) with measures of dietary intake at 14 months and cf-PWV at age 6 years [30]. There was evidence that higher total fat intakes (highest vs. lowest tertile) at 14 months were associated with increased cf-PWV at 6 years (e.g., adverse effect), and total carbohydrate and mono-and disaccharides (middle vs. lowest tertile) were inversely associated with cf-PWV (e.g., protective effect). However, there were no other associations with total protein or saturated fat intake, nor other sub-components of these macronutrients (e.g., vegetable and animal protein, mono and polyunsaturated fat or polysaccharides) (models adjusted for maternal and child-related covariates, including energy intake) [30].

##### Cross-Sectional Studies

One cross-sectional study assessed the relationship between macronutrient intake and arterial stiffness [31]. In this study of adolescents with overweight or obesity (*n* = 183), there was no association between the percentage of total energy from fat intake and cf-PWV and a positive association between the percentage of energy from protein intake and cf-PWV; however, consumption of neither macronutrient was associated with AIx (adjusted for child-related covariates) [31]. In addition, one prospective cohort study reported a positive cross-sectional association between the percentage of energy from fat intake and PWV at 10 years of age [28].

#### 3.5.7. Sodium Intake

##### Cross-Sectional Studies

One cross-sectional study examined the association between sodium intake and arterial stiffness [16]. Brady et al. reported associations between sodium density (mg/kcal/d) and three indexes of arterial stiffness (cf-PWV, AIx, brachial artery distensibility) in a sample of USA participants (*n* = 614), which included children and young adults (10–24 years, mean age 17.9 years) [16]. With a higher sodium diet, a significant inverse association was reported for brachial artery distensibility (adverse effect) and positive associations for carotid-femoral-PWV and AIx (both adverse effects) (adjusted for child-related covariates, including body fat and systolic blood pressure).

**Figure 2:**
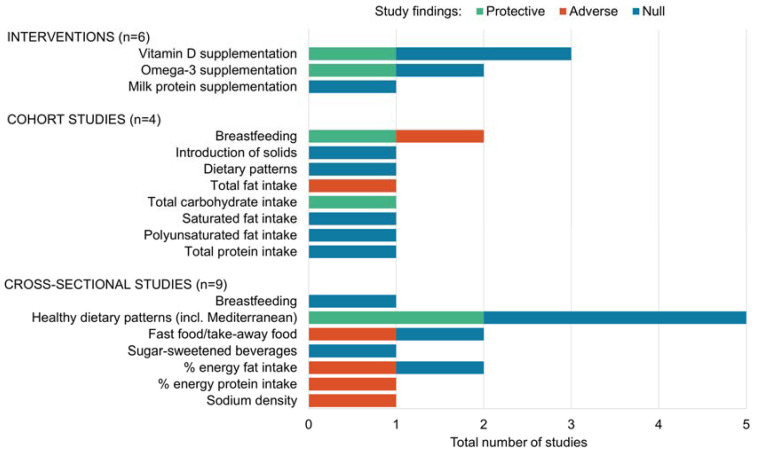
Summary of the associations reported between dietary factors and arterial stiffness in children and adolescents.

Figure 2 summarizes the association between the various dietary factors and markers of arterial stiffness examined in this review.

## 4. Discussion

This systematic review is the first to examine the association between diet and arterial stiffness in children and adolescents. Overall, the evidence across the range of dietary factors examined in this review was scarce. There was limited evidence to suggest a protective effect of vitamin D and omega-3 supplementation and a healthy dietary pattern on arterial stiffness in the pediatric population versus an adverse effect from total fat intake, sodium intake and fast-food consumption. A lack of significant findings across intervention and longitudinal studies may be reflective of the longer time period required for meaningful vascular remodelling to occur and induce changes to arterial stiffness [38]. Overall, the clinical implications of the observed effects on arterial stiffness are unclear, as the overall effect estimates were generally small and may not be clinically relevant. Nonetheless, a previous systematic review found that a relatively small 1 m/s increase in PWV was associated with a 15% increase in cardiovascular mortality and a 14% increase in overall CVD events in adults [6]. Although the implications of increased arterial stiffness in the pediatric population on future cardiovascular outcomes are largely unexplored, relatively small changes in PWV during childhood may still hold relevance for future cardiovascular health.

### 4.1. Intervention Studies

Overall, there were very few intervention studies examining the effect of dietary intervention on arterial stiffness in children, and all of these were based on single-nutrient dietary supplementation. The effect of vitamin D supplementation on arterial stiffness was inconsistent, with two studies showing no effect in an overweight/obese population and a single study in African-American youth finding a significant improvement in cf-PWV, the gold standard measure for arterial stiffness, following supplementation [8,9]. The mechanism for these vasculoprotective effects may be attributed to an array of direct or indirect actions on vascular cells, such as suppression of the renin-angiotensin system, impacts on calcium metabolism and reducing inflammation and oxidative stress on cells [23]. However, the magnitude of this decrease in cf-PWV observed post-supplementation was small (~0.08 m/s) and may not be clinically significant when considering implications on future CVD risk [6]. Furthermore, black youth are at increased risk of vitamin D deficiency in comparison to the white population; Dong et al. [39] previously found that compared to white subjects, Black youth had significantly lower plasma 25-hydroxyvitamin D levels in every season of the year. Thus, the favourable outcomes seen in the Black youth cannot be extrapolated to the broader pediatric population, as these underlying physiological differences may impact outcomes.

The evidence for omega-3 supplementation is inconclusive, with several limitations observed in these studies. Based on evidence from two lower-quality studies, one detected no changes in brachial-PWV, arterial structure or function in a 3-year follow-up of an intervention in young children [27]. A major limitation of this study was that it did not consider potential confounding diet and lifestyle factors between the cessation of interventions and the 3-year follow-up measurements. Another RCT found no significant effects on cr-PWV post-supplementation; however, a reduction in AIx was observed [26]. Comparability and validity of these studies are limited due to variations in sites measured for PWV and the overall lack of use of the gold-standard outcome measure for PWV (e.g., cf-PWV). A previous systematic review in adults found that chronic omega-3 supplementation between six weeks and two years in duration was effective in reducing PWV and improving arterial compliance [12]. Nevertheless, such differences may be indicative of the physiological differences based on age; thus, further studies of long-term omega-3 supplementation in children that utilize the gold standard outcome measure of cf-PWV are warranted to determine if a beneficial effect may be seen in the pediatric population.

### 4.2. Observatoinal Studies

The effect of a range of dietary patterns on arterial stiffness has been investigated in children. Most of these have been cross-sectional studies, with very limited evidence from higher-quality, prospective study designs. The most frequently studied dietary exposure across cross-sectional studies was dietary patterns (Figure 1). The overall evidence for associations between dietary patterns and arterial stiffness was mixed, with the majority reporting null effects [17,34,35]. Furthermore, evidence from the one well-designed prospective cohort study assessing dietary pattern trajectories during early to middle childhood found no association with changes in cf-PWV measured in adolescence [18]. Among adults, a Mediterranean diet has been associated with improved arterial stiffness in adult populations [40]. It is thought that the high fruit, vegetable, nut, fish and olive oil content of the Mediterranean diet improves vascular measures due to their antioxidant effect, thus reducing oxidative stress [41,42]. Further studies in children are required to determine the potential effect of healthy dietary patterns, such as the Mediterranean diet, on arterial stiffness in the pediatric population.

Limited evidence exists for associations between various food groups and arterial stiffness. One study found that from 14 different food groups, the only association with cf-PWV was with fast food intake, suggesting a detrimental effect [17]. On the contrary, Saraf et al. [37] reported no association between the consumption of takeaway foods or sugar-sweetened beverages and cf-PWV.

Two studies examined the relationship between dietary macronutrient composition and arterial stiffness, with some evidence to suggest a relationship between these factors, although the magnitude of these associations was small, raising questions of clinical significance [30,31]. Higher total fat intakes were associated with increased arterial stiffness in one well-designed cohort study with a long six-year follow-up period [30]. A possible mechanism for this relationship is higher intakes of fat, particularly saturated fatty acids, being associated with increased plasma LDL-cholesterol concentrations [43], which result in vascular remodelling and may initiate the underlying processes of atherosclerotic plaque formation, suspected to begin during childhood and progress later into life [44]. However, this same study [30] found no association between different types of fat (including saturated fat) and PWV. Further studies are required to clarify the relationship between different types of fat intake and arterial stiffness within the pediatric population.

A relationship was also evident between arterial stiffness and carbohydrate intake, with significant improvements in arterial stiffness seen at moderate-high intake, compared to low intake [30]. However, the effect size of these findings was small and does not provide information on carbohydrate sources or overall diet quality. Additionally, this study is limited as habitual dietary intake may not be represented due to the single-timepoint dietary assessment and potential confounding dietary changes over the 6-year follow-up period were not considered.

One small, cross-sectional study reported a positive relationship between percentage energy intake from protein and PWV in a cohort of children with habitual low milk intake [31]. The study attributed the detrimental effect of protein to high meat consumption; however, it did not report on the type of protein consumed. Given the variability in health effects of different protein sources, the quality of protein is important to consider [45]. The study also reported a trend for an inverse association between milk intake and PWV (i.e., protective) [31]; however, an RCT in the same cohort found no effect of milk proteins on arterial stiffness [24]. The 12-week intervention period of the RCT may not have been long enough to produce a meaningful result. Milk is expected to play a protective role in vascular health due to its high essential amino acid, vitamin and mineral content and the role of these in protein synthesis and fatty acid oxidization; however, further research is warranted to examine the potential effect on markers of arterial stiffness among children [46].

There was limited evidence examining the relationship between breastfeeding and arterial stiffness in childhood, with two cohort studies of varying quality showing inconsistent results. Overall, the evidence presented in this review is not strong enough to infer that a relationship exists between breastfeeding and arterial stiffness due to both a small sample size in one study [28] and the potential for residual childhood dietary confounding factors that were not adjusted for in analysis [28,29]. Furthermore, breastfeeding is an integral part of dietary guidelines and has been well established to have a range of health benefits, including reduced risk of developing CVD risk factors, such as obesity, high blood pressure and type 2 diabetes [47,48].

Only one study has investigated the association between salt intake and arterial stiffness in children with findings consistent with those in the adult population, with lower sodium intake associated with protective effects on a range of arterial stiffness markers [16]. Salt intake has previously been shown to be detrimental to endothelial function and arterial stiffness in normotensive and hypertensive individuals [14,49]. Salt has been found to have these vascular effects independent of blood pressure, making it highly relevant for a young, healthy population [12,50]. Its effect is potentially due to high salt intake increasing the generation of reactive oxygen species, which thereby reduces the stimulation of nitric oxide, a potent vasodilator [51]. Further studies that include long follow-up periods into adolescence and young adulthood are required to confirm these findings.

### 4.3. Clinical Implications and Future Research

From the current systematic review, limited evidence suggested that some pediatric dietary factors may modulate arterial stiffness; these tended to be in line with general healthy eating patterns and dietary guidelines. In particular, higher total fat and salt intake and less healthy dietary patterns overall were linked to increased arterial stiffness. These relationships are in line with general dietary recommendations, which emphasize the importance of limiting total fat intake and minimizing intake of discretionary foods that are high in fat and salt in order to reduce the risk of various adverse health outcomes, such as overweight/obesity and CVD [48,52,53].

However, given the thin spread of evidence across all study designs for associations between diet and arterial stiffness in children, further research is required to confirm any potential associations found in the current review. Previous studies outside the scope of this review have examined the relationship between childhood dietary factors and arterial stiffness outcomes later into adulthood. A recent large cohort study found no correlation between childhood calcium intakes and PWV when measured in later adulthood [54]. However, another large cohort study by Aatola et al. [38] that examined the relationship between lifetime risk factors and its implications on PWV in adulthood found that persistently higher consumption of fruit and vegetables in childhood and adulthood was associated with lower adulthood PWV, thereby displaying a protective relationship between arterial stiffness and fruit and vegetable consumption. Although the implications of increased arterial stiffness in the pediatric population on future cardiovascular outcomes are largely unexplored, evidence of an association with target organ damage in children [7] alongside the potential impact of childhood dietary risk factors on arterial stiffening and CVD risk later in life [6,7,55] provides a strong rationale for further research in this area.

Given the strong findings in adult populations for the roles of omega-3 supplementation and salt intake on modulating arterial stiffness, it is suggested that future research focuses on these dietary interventions [12,56]. High-quality randomized controlled trials for single nutrient interactions such as omega-3 and salt intake are recommended to ascertain if a causal effect on arterial stiffening exists in the paediatric population. Furthermore, well-designed cohort studies, such as those assessing a range of dietary patterns during the formative childhood years with longer follow-up periods into adulthood, such as that seen in the Young Finns study [38,54], are recommended to allow for meaningful changes in vascular structure and function to occur. Additionally, a standardized measure of arterial stiffness, cf-PWV, should be used as this has been recommended in the paediatric population [9,55].

### 4.4. Strength and Limitations

Research on this topic is not without limitations. Firstly, heterogeneity amongst dietary variables, study designs and measures of arterial stiffness made it difficult to synthesise results. This, combined with a low number of included studies, meant a meta-analysis could not be performed. Not all studies employed the reference method for arterial stiffness measurement (cf-PWV) [9,57]. Furthermore, this review only included research with participants with a population mean age of <18 years. Although results are specifically relevant to the paediatric population, significant findings have been reported in studies of greater duration beyond childhood and adolescent years [38], implying that a longer follow-up period may be required to observe the impact of dietary intake changes during childhood on arterial stiffness in adulthood. As the review was limited to the inclusion of studies published in English only, studies published in languages other than English may have been overlooked.

In terms of the study methodology of included research, several limitations were identified. Significant variables remained unadjusted for in multiple studies, including physical activity [34] and cardiorespiratory markers [18], which have been shown to impact arterial stiffness [58]. Three studies assessing the effect of breastfeeding on arterial stiffness in later childhood had a follow-up period between six and ten years, with dietary measures unaccounted for between infancy and follow-up [28,29,33]. Inconsistency in outcome measurement occurred in three studies, with arterial stiffness not measured in all participants included in final analyses, reducing the validity of the data presented [24,27,28]. Multiple observational studies reported a small sample size [18,28,31,34]. The overarching tools used for dietary analysis were food frequency questionnaires and dietary recall, leading to the possibility of recall bias or social desirability bias, in which participants will report data thought to be more ‘desirable’ by society [59]. However, objective measurement of diet is near impossible for cohort studies analysing usual dietary habits. Overall, the quality of research included in this review is not exceptional, with a significant number of studies reporting insubstantial information on withdrawals, poor participant accountability, biased participant selection and/or unblinded approaches to variable measurement, increasing the risk of bias. However, the majority of included studies employed valid and reliable outcome measures and all studies reported no conflict of interest.

This systematic review has several strengths. The PRISMA guidelines were followed throughout the review process. This review is strengthened by a comprehensive search strategy and data extraction process. A search strategy encompassing a broad range of search terms was developed and used across five databases to obtain relevant articles. Data extraction and quality assessment were completed in duplicate to minimise the risk of bias. Data extraction was piloted, and amendments were made to ensure that all relevant information was captured from the included research.

## 5. Conclusions

This systematic review found that various dietary exposures may play a role in mediating arterial stiffness in a paediatric population. Although evidence of single nutrient interactions from intervention trials was limited, evidence from observational studies indicated that higher total fat and salt intakes may be associated with increased arterial stiffening in children. However, the strength of this evidence is inconsistent, methodologies are varied and only a small number of studies have been conducted in this area. Given the evidence for meaningful impacts of various dietary exposures on modulating arterial stiffness in adults, more robust studies in the paediatric population that implement longer follow-up periods are warranted to ascertain any potential associations and ensure that sufficient time is provided for vascular remodelling to induce meaningful changes in arterial stiffness.

## Figures and Tables

**Table 1:** Summary of characteristics of included studies (*n* = 19).

Characteristic	No. of Studies
Study design	
Intervention	6
Prospective cohort	4
Cross-sectional	9
Dietary exposure	
Supplemental vitamin D	3
Supplemental omega-3 fatty acids	2
Supplemental milk proteins	2
Infant feeding practices	3
Dietary patterns	6
Macronutrient intake	2
Consumption of specific food groups	3
Sodium	1
Outcome: index of arterial stiffness	
PWV	18
Cf-PWV	17
Carotid-radial PWV	3
Carotid-distal PWV	1
Radial-distal PWV	1
Carotid-dorsalis-pedis (foot) PWV	1
Brachial PWV	1
AIx	7
Arterial-AIx	5
Radial-AIx	2
Carotid arterial distensibility	1
Brachial artery distensibility	1

PWV: pulse wave velocity, AIx: Augmentation index.

**Table 2:** Characteristics of included intervention studies (*n* = 6).

Citation	Study Design	Location and Name of Study (If Any)	Sample ^1^	Age (Mean (SD) Unless Stated)	% Male	Weight Status or If Not Reported, BMI	Index of Arterial Stiffness	Diet Component
Arnberg et al., 2013 [24]	Parallel RCT	Copenhagen, Denmark	*n* = 173 Aged 12–15 years, overweight or with obesity, consuming ≤250 mL/d milk and yoghurt, recruited via Civil Registration System	Males: 13.2 (0.7) years Females: 13.2 (0.7) years	38%	Median BMI 25.0 kg/m^2^	Cf-PWV Radial AIx (Applanation tonometry, SphygmoCor System, AtCor Medical)	Milk, e.g., skimmed milk (casein: whey ratio of 80:20) and milk proteins, e.g., whey-based drink (casein: whey ratio of 0:100) and casein-based drink (casein: whey ratio of 100:0) All drinks contained 35 g/100 g total protein
Rajakumar et al., 2020 [19]	Parallel RCT	Pittsburgh, Pennsylvania, US	*n* = 225 Aged 10–18 years, overweight or with obesity, with vitamin D deficiency (serum 25(OH)D < 20 ng/mL), recruited from primary care centre of children’s hospital and advertisements on paediatric research network	13.6 (2.3) years	35%	Overweight 33.3% Obese 66.7%	Cf-PWV Aortic AIx-75 (Arterial tonometry, SphygmoCor CVMS V9 CPVH System, AtCor Medical)	Supplemental vitamin D3
Varshney et al., 2019 [25]	Parallel RCT	India	*n* = 189 Aged 11–17 years with obesity, recruited from endocrinology clinic for management of obesity	Intervention: 12.9 (1.6) years Control: 13.2 (1.5) years	64%	Obese 100%	Cf-PWV Carotid-radial PWV Carotid-distal PWV Radial-distal PWV (Applanation tonometry, SphygmoCor, AtCor Medical)	Supplemental vitamin D
Dong et al., 2010 [23]	Parallel RCT	Richmond County, Augusta, Georgia, US	*n* = 35 Aged 14–18 years, apparently healthy African-American adolescents recruited from high schools	Intervention: 16.5 (1.4) y Control: 16.3 (1.1) years	Intervention: 44% Control: 71%	Mean (SD) BMI percentile: Experimental: 67.8 (SD 30.9) Control: 61.6 (33.4)	Cf-PWV Carotid-radial PWV Carotid-dorsalis-pedis (foot) PWV (Applanation tonometry, Millar Instruments with SphygmoCor software, AtCor Medical)	Supplemental vitamin D3
Dangardt et al., 2010 [26]	Randomized Cross-over trial	Sweden	*n* = 25 Aged 14–17 years with obesity, referred to outpatient clinic for obesity treatment	15.7 (1.0) years	44%	Obese 100%	Carotid-radial PWV (Applanation tonometry, SphygmoCor, AtCor Medical) AIx (Arterial tonometry, Endo-PAT, Itamar Medical Ltd.)	Supplemental omega-3 fatty acid
Ayer et al., 2009 [27]	3-y follow-up of 5-y parallel RCT	Sydney, Australia Childhood Asthma Prevention Study (CAPS)	*n* = 386 Aged 8 years, previously enrolled in 5-years RCT at birth	Intervention: 8.0 (0.1) years Control: 8.0 (0.1) years	Intervention: 50% Control: 52%	Intervention: 17.8 (SD) 3.3 kg/m^2^ Control: 17.4 (2.8) kg/m^2^	Brachial PWV (Electrocardiogram-gated left carotid and radial waveforms) (*n* = 276) Radial AIx-75 Carotid AIx-75 (Applanation tonometry, SPC-301 Millar Instruments with SphygmoCor, AtCor Medical) Carotid arterial distensibility (ultrasound)	≈1:5 ratio of omega-3 to omega-6 fatty acids via supplements and spreads

^1^ Number of participants with 1+ arterial stiffness measurement. AIx: augmentation index; AIx-75: augmentation index normalized to heart rate of 75 bpm; BMI: body mass index; DHA: docosahexaenoic acid; EPA: eicosapentaenoic acid; ITT: intention-to-treat; PWV: pulse wave velocity.

**Table 3:** Characteristics of included observational studies (*n* = 13).

Citation	Study Design	Location and Name of Study (If Any)	Sample ^1^	Age (Mean (SD) Unless Stated)	% Male	Weight Status or If Not Reported, BMI	Index of Arterial Stiffness	Diet Component
**Prospective cohort studies**								
Schack-Nielsen et al., 2005 [28]	Prospective cohort 10 y follow-up (birth to age 10 year)	Copenhagen, Denmark Copenhagen Cohort Study on Infant Nutrition and Growth	*n* = 93 Aged 10 years, recruited from birth cohort study	10.0 (0.1) year	47%	Males Underweight or healthy 90.9% Overweight 9.1% Females Underweight or healthy 91.8% Overweight 8.2%	Cf-PWV Carotid-radial PWV (Optical method, ECG and infrared transducer with Pulse Analysis version 97.1.1 software)	Duration of breastfeeding, current % energy from total fat
de Jonge et al., 2013 [29]	Prospective cohort 6 y follow-up (birth to age 6 years)	Rotterdam, The Netherlands Generation R Study	*n* = 4024 Aged 6 years, mothers enrolled in birth cohort study during prenatal care visit	Ever breastfed: median 6.0 (95% range 5.6–7.4) years Never breastfed: 6.0 (5.6–7.2) years	Ever breastfed: 50% Never breastfed: 49%	Not reported	Cf-PWV (automatic Complior SP device, Artech Medical)	Ever breastfed, breastfeeding duration, breastfeeding exclusivity, age at introduction of solids
Kerr et al., 2018 [18]	Prospective cohort 9 year follow-up (ages 4 y to 15 years)	Melbourne, Australia Parent Education and Support (PEAS) Kids Growth Study	*n* = 188 Aged 15 year, first-born children, parents recruited during routine appointments with Maternal and Child Health Nurses	15.1 (0.5) years	48%	Mean (SD) BMI z-score 0.4 (0.9)	Cf-PWV (Applanation tonometry, SphygmoCor XCEL, AtCor Medical)	4 empirically derived trajectories of dietary patterns
Van den Hooven et al., 2013 [30]	Prospective cohort 5 y follow-up (ages 14 m to 6 years)	Rotterdam, The Netherlands Generation R Study	*n* = 2427 Aged 6 years, mothers enrolled in population-based prospective cohort study	Median 5.9 (95% range 5.6–6.6) years	46%	16.0 (1.6) kg/m^2^	Cf-PWV (automatic Complior SP device, Artech Medical)	Macronutrient intake (total and type)
**Cross-sectional studies**								
Montero López, Mora-Urda, Mill, Silva, Santos Batista and B Molina [33]	Cross-sectional	Vitória, Brazil; Madrid, Spain; Luanda, Angola	*n* = 520 (Brazil *n* = 231, Spain *n* = 176, Angola *n* = 113) Aged 9–10 years, enrolled in elementary schools	Brazil: 9.6 (0.5) years Spain: 9.4 (0.5) years Angola: 9.4 (0.5) years	Brazil: 50% Spain: 53% Angola: 48%	Brazil Underweight or healthy 59.9% Overweight or obese 45% Spain Underweight or healthy weight 61.4% Overweight or obese 38.6% Angola Underweight or healthy weight 83.2% Overweight or obese 16.8%	Cf-PWV (applanation tonometry, SphygmoCor System, AtCor Medical)	Duration of exclusive breastfeeding
Saeedi et al., 2020 [35]	Cross-sectional	Dunedin, New Zealand Physical Activity, Exercise, Diet, And Lifestyle Study (PEDALS)	*n* = 389 Students in years 5 or 6 of primary school, recruited from 17 schools	9.7 (0.7) years	49%	Underweight or healthy weight 81.2 Overweight 14.2% Obese 4.6%	Cf-PWV AIx-75 (site unclear) (Applanation tonometry, SphygmoCor XCEL, AtCor Medical)	2 empirically derived dietary patterns
Lydakis et al., 2012 [32]	Cross-sectional	Heraklion, Crete, Greece	*n* = 277 Students in first year of high school, recruited from three schools	12 (8) years	48%	Healthy 56.7% Overweight 30.3% Obese 13.0%	Arterial AIx (Oscillometric method, PulseCor)	Mediterranean dietary pattern
Ruiz-Moreno et al., 2020 [34]	Cross-sectional	Malaga, Spain	*n* = 75 Aged 6–11 years, recruited from preschools and elementary schools, BMI in ≥95th percentile for age and sex	10.1 (1.3) years	54%	Obese 100%	Cf-PWV (simultaneous tonometry using oscillometric device, Vicorder, Skidmore Medical LTD)	Mediterranean dietary pattern
Pucci et al., 2021 [36]	Cross-sectional	Terni, Italy MACISTE (Metabolic And Cardiovascular Investigation at School, TErni) Study	*n* = 387 Aged 13–19 years, recruited from one school	17.1 (1.4) years	53%	Underweight or healthy 88% Overweight 11% Obese 1%	Cf-PWV (Applanation tonometry, SphygmoCor Vx, AtCor Medical)	Heart-healthy dietary pattern
Giontella et al., 2019 [17]	Cross-sectional	Verona South, Italy	*n* = 300 Aged 7–10 years, recruited from four primary schools	8.6 (0.7) years	50%	Male Healthy 63.3% Overweight 23.3% Obese 13.4% Female Healthy 68% Overweight 19.3% Obese 12.7%	Cf-PWV (applanation tonometry, SphygmoCor XCEL, AtCor Medical)	Intake of 14 foods/food groups and 2 empirically derived dietary patterns
Saraf et al., 2022 [37]	Cross-sectional	Australia Child Health CheckPoint (sub study of the Longitudinal Study of Australian Children (LSAC))	*n* = 1780 Aged 11–12 years, recruited from population-based birth cohort study	11.5 (0.5) years	51%	Underweight or healthy 77.1% Overweight 18.2% Obese 4.8%	Cf-PWV (applanation tonometry, SphygmoCor XCEL, AtCor Medical)	Consumption of fast food and sugar-sweetened beverages
Arnberg et al., 2012 [31]	Cross-sectional	Copenhagen, Denmark	*n* = 183 Aged 12–15 years, overweight or with obesity, consuming ≤250 mL/d milk and yoghurt, recruited via Civil Registration System for participation in intervention study [24]	13.2 years	38%	25.0 kg/m^2^	Cf-PWV AIx-75 (Applanation tonometry, SphygmoCor System, AtCor Medical)	Energy intake, % energy from fat, % energy from protein, milk and yoghurt intake
Brady et al., 2022 [16]	Cross-sectional	Cincinnati, US	*n* = 614 Aged 10–24 years, 31% with type 2 diabetes, 32% with obesity, enrolled in study examining effect of obesity and T2DM on cardiovascular function	17.9 (3.3) years	35%	Underweight or healthy 36.9% Overweight 31% Obese 32.1%	Cf-PWV AIx-75 (applanation tonometry, SphygmoCor SCOR-PVx System above, AtCor Medical) Brachial artery distensibility (DynaPulse Pathway System, Pulse Metric Inc.)	Sodium intake, sodium density

^1^ Number of participants with 1+ arterial stiffness measurement. AIx: augmentation index; AIx-75: augmentation index normalized to heart rate of 75 bpm; BMI: body mass index; DHA: docosahexaenoic acid; EPA: eicosapentaenoic acid; ITT: intention-to-treat; PWV: pulse wave velocity.

**Table 4:** Findings from intervention studies (*n* = 6).

Citation	Study Design	Dietary Component	Intervention	Duration	Blinding and Intention-to-Treat	Main Findings (Effect of Diet Component on A/S) O No Association ↑ Increased ↓ Decreased	Details of Findings	Study Quality ^1^
Arnberg et al., 2013 [24]	Randomized parallel intervention	Consumption of skimmed milk, whey drink or casein drink	1 L/d (containing 35 g/L protein) skimmed milk, whey drink or casein drink vs. water or pre-test control	12-w for the milk-based drinks Subgroup of participants had an additional 12-w as the pre-test control group	Investigators, skimmed milk, whey and casein groups blinded; water and pre-test control groups unblinded Yes ITT	Skimmed milk, whey drink and casein drink: O cf-PWV O radial-AIx	No difference in cf-PWV or radial-AIx in the skimmed milk, whey or casein groups relative to the water group	(Ø)
Rajakumar et al., 2020 [19]	Parallel RCT	Supplemental vitamin D3	2000 vs. 1000 vs. active control 600 IU/d vitamin D3	6-m	Double-blinded Yes ITT	1000 IU and 2000 IU (vs. 600 IU): O cf-PWV; O AIx	No difference in cf-PWV or aortic-AIx in the 2000 or 1000 IU/d groups relative to the 600 IU/d group	(+)
Varshney et al., 2019 [25]	Parallel RCT	Supplemental vitamin D	120,000 vs. active control 12,000 IU/d vitamin D (form not specified)	1 year	Double-blinded No ITT	120,000 IU (vs. 12,000 IU): O cf-PWV; O cr-PWV; O cd-PWV; O rd-PWV	No difference in cf-, cr-, cd- or rd-PWV between the 120,000 or 12,000 IU/d groups	(Ø)
Dong et al., 2010 [23]	Parallel RCT	Supplemental vitamin D3	2000 vs. active control 400 IU/d vitamin D3	16-w	Investigators blinded; participants unblinded No ITT	2000 IU/d: ↓ cf-PWV O cr-PWV O carotid-dorsalis-pedis (foot) PWV	Significant time by group effect: cf-PWV decreased after 16-w of 2000 IU/d (baseline 5.41 ± 0.73, 16-w 5.33 ± 0.79 m/s) and increased after 16-w on 400 IU/d (5.38 ± 0.53, 16-w 5.71 ± 0.75 m/s; interaction *p* = 0.016) No difference in cr-PWV or carotid-dorsalis-pedis PWV between the 2000 and 400 IU/d groups	(+)
Dangart et al., 2010 [26]	Cross-over trial	Supplemental omega-3 fatty acid	Total omega-3 1.2 g (930 mg EPA + 290 mg DHA + 100 mg GLA + 18 mg vitamin E per day) vs. placebo capsules (medium chain triglycerides)	3-m in each condition with 6-w washout	Double-blinded No ITT	O cr-PWV ↓ AIx	No difference in cr-PWV between supplemental and placebo conditions AIx was lower following omega-3 condition (−15.0 ± 7.6%) compared to following the placebo condition (−11.4 ± 11.2%; *p* = 0.05) Negative correlations between ∆AIx and ∆serum EPA (r = −0.47, *p* = 0.025) and ∆AIx and ∆total serum omega-3 fatty acids (r = −0.47, *p* = 0.02)	(Ø)
Ayer et al., 2009 [27]	3-y follow-up of 5-y parallel RCT	≈1:5 ratio of omega-3 to omega-6 fatty acids via supplements and spreads Plasma EPA, DHA, ALA, LA, AA	≈1:5 ratio of omega-3 to omega-6 fatty acids via supplements and oils/spreads vs. control ratio found in general population (range 1:15–1:20)	5-y intervention, 3-y follow-up	Double-blinded Yes ITT	1:5 ratio: O b-PWV O r-AIx O c-AIx O CAD	No difference in b-PWV, CAD, r-AIx-75 or c-AIx-75 between 1:5 group and control group 3-y post-intervention Pearson correlation coefficients between markers of arterial stiffness at 8 years and plasma concentrations of specific omega-3 (DHA, EPA, ALA) and omega-6 (LA, AA) fatty acids measured at 18 mo, 3 y and 5 y 18 mo: No association between any omega-3 or omega-6 fatty acids and b-PWV, CAD, r-AIx-75 or c-AIx-75 3 y: No association between any omega-3 or omega-6 fatty acids and b-PWV, CAD, r-AIx-75 or c-AIx-75 5 y: weak correlation between plasma EPA and b-PWV (−0.15, *p* = 0.04) and between r-AIx-75 and linoleic acid (r = 0.14, *p* = 0.01).	(+)

^1^ Study quality as determined by Academy of Nutrition and Dietetics Quality Criteria Checklist [22]. AA: Arachidonic acid; AIx: augmentation index; c-AIx: augmentation index at the carotid artery; r-AIx: augmentation index at the radial artery; ALA: alpha-linolenic acid; b-PWV: brachial PWV; CAD: carotid artery distensibility; cf-PWV: cf-PWV; cr-PWV: carotid-radial PWV; DHA: docosahexaenoic acid; EPA: eicosapentaenoic acid; GLA: gamma-linolenic acid; ITT: intention-to-treat; LA: linoleic acid; PWV: pulse wave velocity.

**Table 5:** Findings from observational studies (*n* = 13).

Citation	Study Design	Diet Component	Diet Assessment Method	Covariates	Main Findings O No Association ↑ Associated with Increase ↓ Associated with Decrease	Details of Findings ^1^	Study Quality ^2^
Schack-Nielsen et al., 2005 [28]	Prospective cohort 10 y follow-up (birth to age 10 y)	Duration of breastfeeding, current % energy from total fat	Breastfeeding status reported at 9 m of age Duration (months) of breastfeeding included exclusive or partial (at least once daily) breastfeeding. Energy and fat intake at age 10 y assessed via 7-d food record completed by parents, children reported food consumed at school.	Gender, height, weight, body fat percentage (DEXA), SBP, DBP, energy, % energy from fat, time spent in physical activity that affected breathing (1-day diary)	Longer duration breastfeeding: ↑ cf-PWV; O cr-PWV Greater current % energy from fat: ↑ cf-PWV; ↑ cr-PWV Greater current total energy intake: O cf-PWV; ↑ cr-PWV	Longer duration of breastfeeding (months) was associated with higher cf-PWV (β-coefficient = 2.1 (95% CI 0.4, 3.7) cm/s, *p* < 0.05). No association between duration of breastfeeding and cr-PWV. Cross-sectional associations at 10 years: Greater current % energy from fat was positively associated with cr-PWV (β-coefficient = 3.1 (95% CI 0.9, 5.2) cm/s, *p* < 0.01) and cf-PWV (β-coefficient = 1.8 (95% CI 0.2, 3.2), *p* < 0.05). Greater energy intake (MJ/d) was negatively associated with cr-PWV (β-coefficient = −6.4 (95% CI −11.7, −0.8) cm/s, *p* < 0.05) but not associated with cf-PWV.	(+)
de Jonge et al., 2013 [29]	Prospective cohort 6 y follow-up (birth to age 6 y)	Ever breastfed, breastfeeding duration, breastfeeding exclusivity, age at introduction of solids	Questionnaires at infant ages 2, 6 and 12 m (medical records were used if responses were missing) Breastfeeding duration: <2 mo, 2- < 4 mo, 4- < 6 mo or ≥6 mo Breastfeeding exclusivity based on age formula and solid food were introduced: never, partial for ≥4 mo or exclusive for ≥4 mo Introduction of solid foods based on age fruit or vegetable snack was given for first time: <4 mo, 4–5 mo, >5 mo	Maternal age, ethnicity, parity, educational level, prepregnancy BMI, smoking during pregnancy and child’s sex, gestational age at birth, birth weight, weight gain between birth and first year of life, current age, body surface area	Ever breastfed (vs never breastfed): ↓ cf-PWV Duration of breastfeeding: O cf-PWV Breastfeeding exclusivity: O cf-PWV Age at introduction of solids: O cf-PWV	cf-PWV at age 6 y was higher in never-breastfed children compared to those who had ever been breastfed (β-coefficient for never-breastfed = 0.13 (95% CI 0.03, 0.24) m/s) No significant associations between cf-PWV and duration or exclusivity of breastfeeding or age at introduction of solids.	(+)
Kerr et al., 2018 [18]	Prospective cohort 9 y follow-up (ages 4 y to 15 y)	4 empirically derived dietary pattern trajectories: healthy, moderately healthy, moderately unhealthy and unhealthy	4-d food checklist (two weekdays and two weekend-days) collected on eight occasions—six times between ages 4–6.5 y, once at age 10 y and once at age 15 y. Parents or children recorded frequency of consumption of 12 food items (including fruit, milk, water, confectionery). Responses were scored against the 2013 Australian Dietary Guidelines and entered into latent class analysis to produce trajectories of dietary patterns.	Maternal education and socioeconomic position when child was 4 y, current child age, sex, pubertal development and percent of time spent in moderate-to-vigorous physical activity (accelerometer) at age 15-y, BMI z-score	Dietary pattern trajectory: O cf-PWV Dietary pattern score: O cf-PWV	No significant associations between cf-PWV at age 15 y and trajectories of dietary patterns No cross-sectional correlations between cf-PWV and dietary pattern score throughout ages 4.5–15 y	(+)
van den Hooven et al., 2013 [30]	Prospective cohort 5 y follow-up (ages 14 m to 6 y)	Macronutrient intake (total and type): total protein (vegetable protein, animal protein), total fat (saturated fat, monounsaturated fat, polyunsaturated fat), total carbohydrate (mono-and disaccharides, polysaccharides)	221-item validated FFQ developed for Dutch children in second year of life	Macronutrient intakes adjusted for energy intake, models further adjusted for maternal educational, smoking during pregnancy, child’s sex, ethnicity, birth weight, television watching in childhood (<1 h/d or ≥1 h/d), current age, current BMI	Total fat intake: ↑ cf-PWV Saturated, monosaturated or polyunsaturated fat intake: O cf-PWV Total carbohydrate: ↓ cf-PWV Mono-and disaccharides: ↓ cf-PWV Total protein intake: O cf-PWV Vegetable or animal protein: O cf-PWV	Highest tertile of total fat intake at age 14 m was associated with higher cf-PWV at age 6 compared to the lowest tertile (β-coefficient difference between tertile 3 and tertile 1 = 0.11 (95% CI 0.02, 0.20) m/s, *p* = 0.01). No association between middle tertile of fat intake and lowest. Middle tertile of total carbohydrate intake at age 14 m was associated with lower cf-PWV at age 6 compared to the lowest tertile (β-coefficient difference between tertile 2 and 1 = −0.14 (−0.22, −0.05) m/s, *p* < 0.01). No association between highest tertile of carbohydrate intake and lowest. Middle tertile of mono- and disaccharide intake at age 14 m was associated with lower cf-PWV at age 6 compared to the lowest tertile (β-coefficient difference between tertile 2 and tertile 1 = −0.12 (−0.20, −0.03) m/s, *p* < 0.01). No association between highest tertile of mono- and disaccharide intake and lowest. No associations between protein (total, vegetable or animal), saturated, monounsaturated or polyunsaturated fat or polysaccharides and cf-PWV.	(Ø)
Arnberg et al., 2012 [31]	Cross-sectional	Energy, % energy from fat, % energy from protein, milk and yoghurt	4-d food record completed by children (three weekdays and one weekend day).	cf-PWV models adjusted for age, gender, mean arterial pressure, Tanner stage, heart rate and BMI AIx models adjusted for age, gender, Tanner stage, height and BMI	Greater % energy from protein: ↑ cf-PWV; O radial AIx Greater milk and yoghurt intake: ↓ cf-PWV; O AIx Total energy: O cf-PWV O AIx % energy from fat: O cf-PWV O AIx	Greater % energy from protein was associated with higher cf-PWV (β-coefficient for % energy from protein = 0.05 (95% CI not reported) m/s, *p* < 0.01) Trend for greater consumption of milk and yoghurt (g/d) associated with lower cf-PWV (β-coefficient = −0.64 (95% CI not reported) m/s, *p* = 0.05) No associations between energy intake or % energy from fat and cf-PWV or AIx	(Ø)
Brady et al., 2022 [16]	Cross-sectional	Sodium density (mg/kcal/d)	3-d diet record including at least one weekend-day.	Age, sex, race, % body fat via DEXA, T2DM, SBP z-score. Height also included in AIx model.	Higher sodium density ↑ cf-PWV ↑ AIx ↓ brachial artery distensibility	Higher sodium density (mg/kcal/day) associated with lower brachial artery distensibility (β-coefficient = −0.58% change/mm Hg, *p* = 0.004); higher PWV (β-coefficient = 0.42 m/sec, *p* < 0.001) and a trend for higher AIx (β-coefficient = 2.27%, *p* = 0.05). Highest (β-coefficient = −1.55 (−2.36, −0.75) m/s, *p* = 0.0002) and middle tertiles (β-coefficient = −0.76 (−1.55, 0.04) m/s, *p* = 0.06) of sodium density associated with higher cf-PWV compared to the lowest tertile. Highest tertile of sodium density vs. lowest tertile was associated with lower AIx (β-coefficent = −16.0 (95% CI −27.32, −4.61)%, *p* = 0.006). Highest tertile of sodium density vs. lowest tertile was associated with lower brachial distensibility (β-coefficient = −0.26 (−0.47, −0.04)% change/mmHg, *p* = 0.02)	(Ø)
Giontella et al., 2019 [17]	Cross-sectional	Intake of 14 foods/food groups: fast food, cereals and tubers, vegetables, fruit, eggs, meat, dairy products, sweets, legumes, fish, nuts, extra virgin olive oil, animal-derived fat, seed oil 2 empirically derived dietary patterns: ‘healthy’ and ‘unhealthy’	61-item validated FFQ completed by child	Age, sex, ethnicity, BMI, energy intake, physical activity score (PAQ-C score)	Greater consumption of fast food: ↑ cf-PWV cereals and tubers, vegetables, fruit, eggs, meat, dairy products, sweets, legumes, fish, nuts, extra virgin olive oil, animal-derived fat, seed oil: O cf-PWV ‘Healthy’ and ‘unhealthy’ dietary patterns: O cf-PWV	Association between fast food and cf-PWV in adjusted analysis (β-coefficient for fast food = 0.337 (95% CI 0.140, 0.534) m/s *p* < 0.01). There were no associations between cereals and tubers, vegetables, fruit, eggs, meat, dairy products, sweets, legumes, fish, nuts, extra virgin olive oil, animal-derived fat, seed oil and cf-PWV. No significant correlations between the ’healthy’ or ‘unhealthy’ dietary patterns and cf-PWV.	(Ø)
Lydakis et al., 2012 [32]	Cross-sectional	Adherence to Mediterranean dietary pattern	16-item KIDMED questionnaire, child reported	Gender, heart rate, peripheral mean pressure, BMI, waist circumference	Greater adherence to Mediterranean diet: ↓ AIx	Higher adherence to Mediterranean diet associated with lower AIx (β-coefficient for KIDMED score = −0.114 (95% CI not reported), *p* = 0.026).	(Ø)
Montero López et al., 2019 [33]	Cross-sectional	Retrospective information on duration of exclusive breastfeeding	Weeks of exclusive breastfeeding (no other liquids or solids except for vitamin drops or syrups, mineral supplements or medications) extracted from each child’s health cards provided by the family	Sex, birth weight, current BMI, SBP	Duration of exclusive breastfeeding: O cf-PWV	No association between duration of exclusive breastfeeding and cf-PWV.	(Ø)
Pucci et al., 2021 [36]	Cross-sectional	Adherence to heart-healthy dietary pattern	24-h recall and validated FFQ, completed by child. Responses scored against cardiovascular recommendations of (1) ≥400 g/d fruits and vegetables, (2) ≥200 g/wk fish; (3) sodium ≤1500 mg/day; (4) sugar-sweetened beverages ≤450 kcal/wk (1 L/wk) and (5) ≥3 serves/d whole grains scaled to a 2000-kcal/d diet	Unadjusted	Consumption of ‘ideal’ heart-healthy dietary pattern: ↓ cf-PWV	Participants with diets scored by researchers as ‘ideal’ for cardiovascular health had lower cf-PWV than those with diets considered ‘not ideal’ (4.57 m/s vs. 4.98 m/s, *p* < 0.05) (unadjusted univariate analysis)	(Ø)
Ruiz-Moreno et al., 2020 [34]	Cross-sectional	Adherence to Mediterranean dietary pattern	14-item FFQ (not reported if parent or child completed)	Age, weight, BMI, BMI z-score, serum total cholesterol, serum LDL-cholesterol, serum HDL-cholesterol, fasting glucose, serum insulin levels, HOMA-IR index	Adherence to Mediterranean diet: O cf-PWV	No association between adherence to Mediterranean dietary pattern and cf-PWV.	(Ø)
Saeedi et al., 2020 [35]	Cross-sectional	2 empirically derived dietary patterns: ‘snacks’ and ‘fruit and vegetables’ identified using principal component analysis	28-item validated FFQ completed by child	Age, sex, ethnicity, socioeconomic deprivation, heart rate, mean arterial pressure, moderate and vigorous physical activity (accelerometer), cardiorespiratory fitness (VO2max), fat mass, fat free mass (bioelectrical impedance analyzer)	‘Snacks’ or ‘fruit and vegetables’ dietary patterns: O cf-PWV; O AIx	No associations between the ‘snacks’ or ‘fruit and vegetables’ dietary patterns and cf-PWV or AIx	(+)
Saraf et al., 2022 [37]	Cross-sectional	Consumption of fast food and sugar-sweetened beverages	FFQ, parent and child reported. Items about fast food and sugar-sweetened beverage consumption dichotomized into <once per week or ≥once per week.	Age, sex, socioeconomic position	Consumption of takeaway food or sugar-sweetened beverages: O cf-PWV	No associations between frequency per week of consumption of takeaway food or sugar-sweetened beverages and cf-PWV	(+)

^1^ β represents unstandardized regression beta-coefficient unless otherwise stated. ^2^ Study quality as determined by Academy of Nutrition and Dietetics Quality Criteria Checklist [22]. AA: arachidonic acid; AIx: augmentation index; ALA: alpha-linolenic acid; AT, applanation tonometry; cf-PWV: cf-PWV; cr-PWV: carotid-radial PWV; HOMA-IR: homeostatic model assessment for insulin resistance; PWV: pulse wave velocity.

## Data Availability

Not applicable.

## Supplementary Materials

The following supporting information can be downloaded at: https://www.mdpi.com/article/10.3390/nu15092092/s1, Supplementary File S1: Table S1: MEDLINE Complete final search strategy (*n* = 2388); Table S2: CINAHL (EBSCO) final search strategy (*n* = 424); Table S3: EMBASE final search strategy (*n* = 1664); Table S4: Scopus final search strategy (*n* = 3046); Table S5: Cochrane Central final search strategy (*n* = 511); Table S6: Quality assessment of studies using the Academy of Nutrition and Dietetics Quality Criteria Checklist (*n* = 19).
