# Supplementary material for: Dietary Intake and Arterial Stiffness in Children and Adolescents: A Systematic Review
Source: Nutrients. 2023 Apr 26;15(9):2092. doi: 10.3390/nu15092092 (PMC10180640; doi:10.3390/nu15092092)
Supplement: Supplementary file 1 [file nutrients-15-02092-s001.zip › nutrients-2294917-supplementary/nutrients-2294917-supplementary.pdf]

Supplementary Table S1. MEDLINE Complete final search strategy (n=2,388)

	Search terms
Concept 1 – keywords	"Dietary intake" OR "diet intake" OR "nutrient intake" OR "nutrient*" OR "food*" OR "food intake*" OR "food consumption" OR "dietary pattern*" OR "dietary quality" OR "dietary diversity" OR "diet" OR “nutrition” OR “vitamin*” OR “mineral*” OR “micronutrient*” OR “macronutrient*” OR “nutrition” OR "sodium" OR “salt” OR “soy*” OR "omega-3" OR “omega-6” OR “polyunsaturated” OR “essential fatty acids” OR “fish oil” OR "fat" OR "vitamin-D" OR "protein" OR "sugar*" OR "carbohydrate*" OR "magnesium" OR "folate" OR “folic acid” OR "olive oil" OR “Mediterranean diet” OR “fiber” OR “fibre” OR “dairy” OR “fruit*” OR “vegetable*” OR “saturated fat” OR “caffeine” OR “polyphenol*” OR “anti-oxidant*” OR “antioxidant*” OR “Plant-based diet” OR “plant based diet*” OR “DASH” OR “alcohol” OR “phytoestrogen*” OR “miso” OR “natto” OR “isoflavone” OR “DASH diet” OR “monounsaturated fat*” OR “unsaturated fat*” OR “vegetarian diet” OR “supplement*” OR “fatty acid*”
	OR
Concept 1 – subject headings	*Diet+ OR Food+ OR Nutrients+ OR Vitamins+ OR Micronutrient+*
	AND
Concept 2 – keywords	TI "arterial stiff*" OR "pulse wave velocity" OR "vascular ageing" OR “vascular aging” OR "PWV" OR "pulse pressure" OR "wave reflections" OR "augmentation index" OR "aortic stiffness" OR “central pressure” OR “arterial compliance” OR “arterial elasticity” OR "intimal medial thickness" OR "IMT" OR “Vascular stiffness” OR “Vascular compliance” OR “Vascular elasticity” OR “Vascular resistance” OR “Arterial elasticity” OR “Arterial resistance” OR “Artery stiffness” OR “Artery compliance” OR “Artery resistance”
	OR
Concept 2 –subject headings	Vascular stiffness
	AND
Concept 3 – keywords	“child*” OR “adolesce*” or “pediatric” OR “paediatric” OR “teen*” OR “young adult*” OR “youth” OR “boy*” OR “girl*”
	OR
Concept 3 –subject headings	Child+ OR Adolescent
	AND
Limits (e.g. publication dates, language).	English, Humans

Supplementary Table S2. CINAHL (EBSCO) final search strategy (n=424)

	**Search terms**
Concept 1 – keywords	"Dietary intake" OR "diet intake" OR "nutrient intake" OR "nutrient*" OR "food*" OR "food intake*" OR "food consumption" OR "dietary pattern*" OR "dietary quality" OR "dietary diversity" OR "diet" OR "nutrition" OR "vitamin*" OR "mineral*" OR "micronutrient*" OR "macronutrient*" OR "nutrition" OR "sodium" OR "salt" OR "soy*" OR "omega-3" OR "omega-6" OR "polyunsaturated" OR "essential fatty acids" OR "fish oil" OR "fat" OR "vitamin-D" OR "protein" OR "sugar*" OR "carbohydrate*" OR "magnesium" OR "folate" OR "folic acid" OR "olive oil" OR "Mediterranean diet" OR "fiber" OR "fibre" OR "dairy" OR "fruit*" OR "vegetable*" OR "saturated fat" OR "caffeine" OR "polyphenol*" OR "anti-oxidant*" OR "antioxidant*" OR "Plant-based diet" OR "plant based diet*" OR "DASH" OR "alcohol" OR "phytoestrogen*" OR "miso" OR "natto" OR "isoflavone" OR "DASH diet" OR "monounsaturated fat*" OR "unsaturated fat*" OR "vegetarian diet" OR "supplement*" OR "fatty acid*"
	OR
Concept 1 – subject headings	*Diet+ OR Food+ OR Vitamins+*
	AND
Concept 2 – keywords	TI "arterial stiff*" OR "pulse wave velocity" OR "vascular ageing" OR "vascular aging" OR "PWV" OR "pulse pressure" OR "wave reflections" OR "augmentation index" OR "aortic stiffness" OR "central pressure" OR "arterial compliance" OR "arterial elasticity" OR "intimal medial thickness" OR "IMT" OR "Vascular stiffness" OR "Vascular compliance" OR "Vascular elasticity" OR "Vascular resistance" OR "Arterial elasticity" OR "Arterial resistance" OR "Artery stiffness" OR "Artery compliance" OR "Artery resistance"
	OR
Concept 2 –subject headings	Arterial stiffness
	AND
Concept 3 – keywords	"child*" OR "adolesce*" or "pediatric" OR "paediatric" OR "teen*" OR "young adult*" OR "youth" OR "boy*" OR "girl*"
	OR
Concept 3 –subject headings	Child+<br>Adolescence+
	AND
Limits (e.g. publication dates, language).	English, Humans

Supplementary Table S3. EMBASE final search strategy (n=1664)

	**Search terms**
Concept 1 – keywords	"Dietary intake" OR "diet intake" OR "nutrient intake" OR "nutrient*" OR "food*" OR "food intake" OR "food consumption" "dietary pattern*" OR "diet*" OR "nutrition" OR "vitamin*" OR "mineral*" OR "micronutrient*" OR "macronutrient*" OR "nutrition" OR "sodium" OR "salt" OR "soy*" "omega-3" OR "omega-6" OR "polyunsaturated" OR "essential fatty acids" OR "fatty acids" OR "fish oil" OR "fat*" OR "vitamin-D" OR "protein*" OR "sugar*" OR "carbohydrate*" OR "magnesium" OR "folate" OR "folic acid" OR "olive oil" OR "Mediterranean diet" OR "fiber" OR "fibre" OR "dairy" OR "fruit*" OR "vegetable*" OR "saturated fat" OR "unsaturated fat*" OR "monounsaturated fat*" OR "caffeine" OR "polyphenol*" OR "anti-oxidant*" OR "antioxidant*" OR "Plant-based diet" OR "plant based diet" OR "vegetarian diet" OR "DASH diet" OR "alcohol" OR "phytoestrogen*" OR "miso" OR "natto" OR "isoflavone" OR "supplement*"
OR	
Concept 1 – subject headings	*Diet/exp OR Food/exp OR Nutrient/exp OR Vitamin/exp OR Mineral/exp OR Dietary Supplement/exp*
AND	
Concept 2 – keywords	"arterial stiff*" OR "pulse wave velocity" OR "vascular ageing" OR "vascular aging" OR "PWV" OR "pulse pressure" OR "wave reflections" OR "augmentation index" OR "aortic stiffness" OR "central pressure" OR "arterial compliance" OR "arterial elasticity" "intimal medial thickness" OR "IMT" OR "Vascular stiffness" OR "Vascular compliance" OR "Vascular elasticity" OR "Vascular resistance" OR "Arterial elasticity" OR "Arterial resistance" OR "Artery stiffness" OR "Artery compliance" OR "Artery resistance"
OR	
Concept 2 –subject headings	*Arterial stiffness/exp*
AND	
Concept 3 – keywords	"child*" OR "adolesce*" or "pediatric" OR "paediatric" OR "teen*" OR "young adult*" OR "youth" OR "boys" OR "girls"
OR	
Concept 3 –subject headings	*Child/exp* OR *Adolescent/exp*
AND	
Limits (e.g. publication dates, language).	English only, no animal studies, no conference abstracts, limited to embase only

Supplementary Table S4. Scopus final search strategy (n=3,046)

	**Search terms**
Concept 1 – keywords	"Dietary intake" OR "diet intake" OR "nutrient intake" OR nutrient* OR food* OR "food intake" OR "food consumption" OR "dietary pattern*" OR diet OR "dietary diversity" OR "dietary quality" OR "nutrition" OR vitamin* OR mineral* OR micronutrient* OR macronutrient* OR sodium OR salt OR soy* OR omega-3 OR omega-6 OR polyunsaturated OR "essential fatty acid*" OR "fatty acid*" OR "fish oil" OR fat OR vitamin-D OR protein* OR sugar* OR carbohydrate* OR magnesium OR folate OR "folic acid" OR "olive oil" OR "Mediterranean diet" OR fiber OR fibre OR dairy OR fruit* OR vegetable* OR "saturated fat*" OR "monounsaturated fat*" OR "unsaturated fat*" OR caffeine OR polyphenol* OR anti-oxidant* OR antioxidant* OR "Plant-based diet" OR "plant based diet*" OR "vegetarian diet*" OR DASH OR "DASH diet*" OR alcohol OR phytoestrogen* OR miso OR natto OR isoflavone OR Supplement*
OR	
Concept 1 – subject headings	*N/A*
AND	
Concept 2 – keywords	pwv OR "arterial stiff*" OR "pulse wave velocity" OR "vascular ageing" OR "vascular aging" OR "wave reflections" OR "augmentation index" OR "aortic stiffness" OR "central pressure" OR "arterial compliance" OR "arterial elasticity" OR "intimal medial thickness" OR imt OR "Vascular stiffness" OR "Vascular compliance" OR "Vascular elasticity" OR "Vascular resistance" OR "Arterial resistance" OR "Artery stiffness" OR "Artery compliance" OR "Artery resistance"
OR	
Concept 2 –subject headings	*N/A*
AND	
Concept 3 – keywords	child* OR adolesce* OR pediatric OR paediatric OR teen* OR "young adult*" OR youth* OR boy* OR girl*
OR	
Concept 3 –subject headings	*N/A*
AND	
Limits (e.g. publication dates, language).	English only, no conference abstracts. Unable to exclude animal studies.

Supplementary Table S5. Cochrane Central final search strategy (n=511)

	Search terms
Concept 1 – keywords	Dietary intake" OR "diet intake" OR "nutrient intake" OR nutrient* OR food* OR "food intake*" OR "food consumption" OR "dietary pattern*" OR "dietary quality" OR "dietary diversity" OR diet OR nutrition OR vitamin* OR mineral* OR micronutrient* OR macronutrient* OR nutrition OR sodium OR salt OR soy* OR omega-3 OR omega-6 OR “polyunsaturated” OR “essential fatty acids” OR “fish oil” OR fat OR vitamin-D OR protein OR sugar* OR carbohydrate* OR magnesium OR folate OR “folic acid” OR "olive oil" OR “Mediterranean diet” OR fiber OR fibre OR dairy OR fruit* OR vegetable* OR “saturated fat” OR caffeine OR polyphenol* OR anti-oxidant* OR antioxidant* OR “Plant-based diet” OR “plant based diet*” OR DASH OR alcohol OR phytoestrogen* OR miso OR natto OR isoflavone OR “DASH diet” OR “monounsaturated fat*” OR “unsaturated fat*” OR “vegetarian diet” OR supplement* OR “fatty acid*
	OR
Concept 1 – subject headings	Diet/exp OR Food/exp OR Nutrient/exp OR Vitamin/exp OR Micronutrient/exp OR Dietary Supplement/exp
	AND
Concept 2 – keywords	"Arterial stiffening" OR “arterial stiffness” OR "pulse wave velocity" OR "vascular ageing" OR “vascular aging” OR PWV OR "pulse pressure" OR "wave reflections" OR "augmentation index" OR "aortic stiffness" OR “central pressure” OR “arterial compliance” OR “arterial elasticity” OR "intimal medial thickness" OR IMT OR “Vascular stiffness” OR “Vascular compliance” OR “Vascular elasticity” OR “Vascular resistance” OR “Arterial elasticity” OR “Arterial resistance” OR “Artery stiffness” OR “Artery compliance” OR “Artery resistance”
	OR
Concept 2 –subject headings	Arterial stiffness/exp
	AND
Concept 3 – keywords	child OR children OR adolescent* OR adolescence OR pediatric OR paediatric OR teenager OR teen OR “young adult*” OR youth OR boy* OR girl*
	OR
Concept 3 –subject headings	Child/exp OR Adolescent/exp
	AND
Limits (e.g. publication dates, language).	Trials + Cochranes automatic limitations

Supplementary Table S6. Quality assessment of studies using the Academy of Nutrition and Dietetics Quality Criteria Checklist (n=19)[1]

Study	Relevance questions				Validity questions										Overall
	1 Outcome is improved	2 Relevant outcome	3 Issue of concern in dietetics	4 Feasible	1 Clear research question	2 Unbiased participant selection	3 Comparable study groups	4 Transparent handling of withdrawals	5 Adequate blinding	6 Description of procedure or exposure	7 Valid and reliable outcome measurement	8 Appropriate statistical analysis	9 Appropriate conclusions	10 No conflict of interest	Risk of bias
**Intervention studies**															
Arnberg 2013	Y	Y	Y	Y	Y	Y	N	Y	N	Y	Y	Y	Y	Y	(Ø)
Ayer 2009	Y	Y	Y	Y	Y	Y	Y	Y	Y	Y	Y	Y	Y	Y	(+)
Dangardt 2010	Y	Y	Y	Y	Y	N	N	N	Y	Y	Y	N	Y	Y	(Ø)
Dong 2010	Y	Y	Y	Y	Y	Y	Y	N	N	Y	Y	Y	Y	Y	(+)
Rajakumar 2020	Y	Y	Y	Y	Y	Y	Y	Y	Y	Y	Y	Y	Y	Y	(+)
Varshney 2017	Y	Y	Y	Y	N	Y	N	Y	N	Y	Y	N	Y	Y	(Ø)
**Prospective cohort studies**															
De Jonge 2013	NA	Y	Y	NA	Y	Y	Y	Y	Y	Y	Y	Y	Y	Y	(+)
Kerr 2018	NA	Y	Y	NA	Y	Y	Y	Y	Y	Y	Y	Y	Y	Y	(+)
Schack-Nielsen 2005	NA	Y	Y	NA	Y	Y	Y	Y	U	Y	Y	Y	Y	Y	(+)
Van Den Hooven 2013	NA	Y	Y	NA	Y	Y	Y	Y	U	Y	Y	Y	Y	Y	(Ø)
**Cross-sectional studies**															
Arnberg 2012	NA	Y	Y	NA	Y	N	N	Y	Y	Y	Y	Y	Y	Y	(Ø)
Brady 2022	NA	Y	Y	NA	Y	N	N	N	Y	Y	Y	Y	Y	Y	(Ø)
Giontella 2019	NA	Y	Y	NA	Y	N	N	Y	Y	Y	Y	Y	Y	Y	(Ø)
Lydakis 2012	Y	Y	Y	NA	Y	N	Y	Y	Y	Y	U	Y	Y	Y	(Ø)
Montero-Lopez 2019	NA	Y	Y	NA	Y	N	N	N	Y	Y	Y	Y	Y	Y	(Ø)
Pucci 2021	NA	Y	Y	NA	Y	N	Y	Y	Y	Y	Y	N	Y	Y	(Ø)
Ruiz-Moreno 2020	Y	Y	Y	Y	Y	N	Y	N	Y	N	Y	N	Y	Y	(Ø)
Saeedi 2020	NA	Y	Y	NA	Y	Y	Y	Y	Y	Y	Y	Y	Y	Y	(+)
Saraf 2022	Y	Y	Y	NA	Y	Y	Y	Y	Y	Y	Y	Y	Y	Y	(+)

Abbreviations: Y, Yes; U, Unclear; N, No; NA, not applicable; (+) positive, indicating low risk of bias; (Ø) neutral, indicating limited risk of bias; (-) negative, indicating high risk of bias

[1]Academy of Nutrition and Dietetics. EVIDENCE ANALYSIS MANUAL: Steps in the Academy Evidence Analysis Process. Chicago: 2016.
